# The Lamp

**Published:** 1849-01

**Authors:** 

**Affiliations:** Xenia, O


### THE LAMP.

BY DR. TAFT.—*Xenia, O.*

An accurate knowledge of small matters in all mechanical pursuits, is an acquisition without which it is impossible to excel, or even to accomplish much. However applicable this may be to any mechanical department, it is equally so to the mechanical dentist. With an imperfect acquaintance with the minutiæ of manipulation, necessary for the accomplished mechanical operator, what *rude specimens* are daily put forth. And one among these small, and by some uncared for, matters, is the soldering lamp. The lamp usually employed by dentists is the large oil lamp. To this lamp there are many objections: the heat or blast from it is not so powerful, and consequently not so efficient, as the spirit lamp; this will at once be apparent, when we consider that the soot or residue of the burning oil is thrown in the blast upon the work; and for this reason the flame from an oil lamp is always sluggish; this soot or residue is more than one-fourth the weight of the oil consumed. The work will become more dirty with the oil than with the spirit lamp; both the lamp and the work soldered is more disagreeable to handle on account of the blackened oil, burned wick, &c.

The spirit lamp would, I believe, be always used, were it not for one or two objections. The most prominent objection to it, is, its liability to explode. A possibility of explosion by the spirit lamp may be precluded by the following construction. It may be made of any capacity; but a lamp that will contain a pint, or pint and a half at most, is sufficiently large for any kind of work upon which it is desirable to use a blow-pipe. It should be made of heavy sheet brass, or German silver, of cylindrical form, about three inches high, and three or four inches in diameter; the top and bottom soldered on perfectly close; through the top, and near one side, a hole about an inch and a quarter in diameter is made, into this is soldered a tube, the lower end of which extends downward in the lamp within one-thirtieth of an inch of the bottom; the upper end about one-half inch above the top of the lamp. The caliber of this tube should be about one inch, for ordinary purposes. Care should be exercised that the soldering of this tube, throughout its whole length, and also in the top of the lamp, be perfect. A coil of iron or steel wire is made that will just drop into this tube, rest upon the bottom, and extend about one-fourth of an inch above the top of the tube; this coil supports the wick, which is put into it. The wick is made large enough to fill the coil, and extends to the bottom of the lamp. A cap is made, which fits close on the top of the tube, and prevents evaporation of the alcohol, when the lamp is not in use. On the opposite side of the lamp, into the top, is soldered a small tube, about half an inch in diameter, for the purpose of filling or emptying the lamp; this tube also has a cap, that fits close upon it, to prevent evaporation.

It will be seen at once, that with this construction, it is impossible for the fire to communicate with the inside of the lamp, and consequently just as impossible that an explosion could take place. Alcohol for the lamp should be ninety or ninety-five per cent. The spirit lamp is not offensive, it makes no smoke, produces no disagreeable fumes, is always clean; it can be used in the operating room, labratory, or any where else that it may be desirable. It serves admirably for warming wax for taking impressions.

